# Simultaneous Improvement of Grain Yield and Quality through Manipulating Two Type C G Protein Gamma Subunits in Rice

**DOI:** 10.3390/ijms23031463

**Published:** 2022-01-27

**Authors:** Lian Wu, Xiaodong Wang, Zhiwen Yu, Xin Cui, Quan Xu

**Affiliations:** Agronomy College of Shenyang Agricultural University, 110866 Shenyang, China; 2019200050@stu.syau.edu.cn (L.W.); 2019220241@stu.syau.edu.cn (X.W.); 2019220263@stu.syau.edu.cn (Z.Y.); 2019500051@syau.edu.cn (X.C.)

**Keywords:** heterotrimeric G protein, rice, gene editing, trait improvement

## Abstract

Heterotrimeric G protein signaling is an evolutionarily conserved mechanism in diverse organisms that mediates intracellular responses to external stimuli. In rice, the G proteins are involved in the regulation of multiple important agronomic traits. In this paper, we present our finding that two type C G protein gamma subunits, *DEP1* and *GS3*, antagonistically regulated grain yield and grain quality. The *DEP1* gene editing we conducted, significantly increased the grain number per panicle but had a negative impact on taste value, texture properties, and chalkiness-related traits. The *GS3* gene editing decreased grain number per panicle but significantly increased grain length. In addition, the *GS3* gene-edited plants showed improved taste value, appearance, texture properties, and Rapid Visco Analyser (RVA) profiles. To combine the advantages of both *gs3* and *dep1*, we conducted a molecular design breeding at the *GS3* locus of a “super rice” variety, SN265, which has a truncated *dep1* allele with erect panicle architecture, high-yield performance, and which is of mediocre eating quality. The elongated grain size of the *sn265/gs3* gene-edited plants further increased the grain yield. More importantly, the texture properties and RVA profiles were significantly improved, and the taste quality was enhanced. Beyond showcasing the combined function of *dep1* and *gs3*, this paper presents a strategy for the simultaneous improvement of rice grain yield and quality through manipulating two type C G protein gamma subunits in rice.

## 1. Introduction

The challenge of meeting the increasing worldwide demand for rice production has driven a sustained quest for advances in rice breeding. China was one of the first places to domesticate and cultivate rice, and today, it is the largest rice-producing country in the world [1]. The last century has witnessed the introduction of semi-dwarf and hybrid rice varieties, leading to quantum leaps in productivity that increased China’s rice yield from 1.9 t ha^−1^ in 1949 to 7.0 t ha^−1^ in 2018 (http://faostat.fao.org/; Data: 12 October 2021). In China’s national new rice variety trial, the average yield of new varieties from 2004 to 2018 reached 9.6 t ha^−1^ [2]. As the economy and living standards have significantly improved during recent decades, studies have focused on rice quality. Eating and cooking quality (ECQ) is one of the most important determinants of grain quality. However, it is challenging to simultaneously improve grain yield and quality.

The heterotrimeric G protein complexes play important roles as signal transducers from receptors to downstream events [3,4,5,6], and the G protein signaling pathways have been extensively studied in both animals and plants because of their role in regulating almost every physiological response [3]. Although the heterotrimeric G protein subunits are all present in plants, the repertoire of G protein in plants is much simpler than that in animals [6]. In the rice genome, single-copy genes encode canonical G alpha (*RGA1*) and G beta (*RGB1*) subunits [7,8,9], and there appear to be two type B G gamma subunits, which do not have canonical isoprenylation domains (*RGG1* and *RGG2*) [10,11]. In addition to the canonical G gamma subunits, the rice genome encodes three type C G gamma subunits, *GS3*, *OsGGC2*, and *DEP1* [6]. Analyses of *DEP1* and *GS3* polymorphisms have revealed their key roles in the regulation of important agronomic traits. Natural variants of the *DEP1* gene were shown to promote panicle branching and improve grain yield. The rice plants harboring a truncated *dep1* allele exhibited increased grain number per panicle, dense and erect panicle architecture, and enhanced grain yield [12,13,14]. Our previous studies demonstrated that more than 50% of rice varieties in the northeast of China carry the truncated allele of *dep1*; moreover, the cultivation area for erect panicle varieties has increased annually in China [13,15]. Unfortunately, the varieties harboring a truncated *dep1* allele are considered to be of mediocre eating and cooking quality, and the short and round grains produced by the truncated *dep1* allele are not favored by consumers. Interestingly, another type C G protein gamma subunit, *GS3*, functions as a negative regulator of grain length [16]. The plants carrying the loss-of-function *gs3* allele produce longer grains than those formed by the functional *GS3* allele [17]. Thus, both of the type C G protein gamma subunits are involved in the regulation of grain yield and quality.

As the two G protein gamma subunits, *DEP1* and *GS3* antagonistically regulate grain size, and the elite allele of each gene compensates for the other’s deficiencies. The aim of this study was to determine whether grain yield and quality could be simultaneously improved through manipulating two type C G protein gamma subunits in rice. In addition, we examined whether *DEP1* and *GS3* were involved in the regulation of other quality traits besides grain shape, such as taste value, texture properties, and Rapid Visco Analyser (RVA) profiles. In this study, we generated the mutant lines of *DEP1* and *GS3* using CRISPR/Cas9 gene-editing technology, and the yield- and quality-related traits were comprehensively investigated. Molecular design breeding was employed for SN265, a high-yield performance variety with erect panicle architecture, to enhance the grain quality through *GS3* gene editing. The study provides a strategy for simultaneous improvement of yield and quality through manipulating two type C G protein gamma subunits in rice. 

## 2. Results

### 2.1. Construction of Two Type C G Protein Gamma Subunit Mutants in Rice

**Figure 1:**
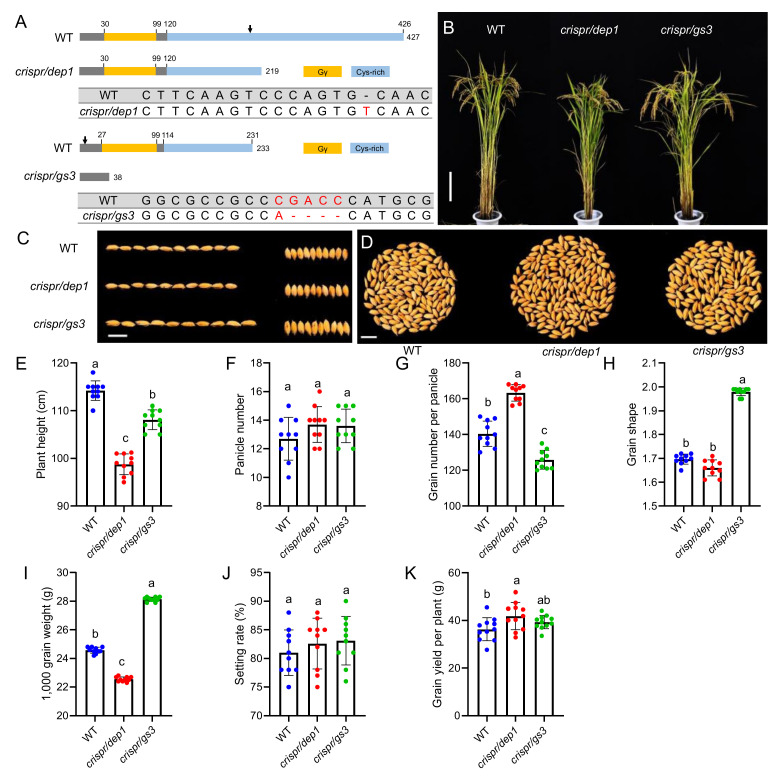
The yield-related traits of the *japonica* variety Sasanishiki (WT) gene-edited mutants. (**A**) Schematic diagram of the genomic region and functional domain of WT and CRISPR gene-edited mutants. The sequence alignment of the sgRNA target region shows altered bases in different lines. The arrows indicate the position of the sgRNA. (**B**) The plant architecture of WT and gene edited mutants. Bar = 20 cm. (**C**) The grain size of WT and gene edited mutants. Bar = 1 cm. (**D**) The grain number per panicle of WT and gene edited mutants. Bar = 1 cm. (**E–K**) The plant height, panicle number, grain number per panicle, grain shape, 1000—grain weight, setting rate, and grain yield per plant of WT and gene edited mutants. The data are the mean ± s.d. (n = 10 plants), and different letters indicate significant differences at the 5% level. The red, blue and green dots indicate the individual values of WT, *crispr/dep1*, and *crispr/gs3*, respectively.

To conduct a comprehensive investigation of the phenotypic changes in the *DEP1* and *GS3* lines, we generated the mutants of two G protein gamma subunits using CRISPR/Cas9 gene-editing technology under the genetic background of the *japonica* rice variety Sasanishiki (WT). *DEP1* has a modular arrangement with a conventional plant-specific G gamma subunit protein domain at its N-terminus, followed by a cys-rich domain at the C-terminus. An allelic investigation of *japonica* rice varieties showed that almost all the erect panicle varieties harbored a truncated *dep1* allele. This type of allele had a 637-bp stretch of the middle of the fifth exon replaced by a 12-bp sequence, which created a premature stop codon and caused loss of the cys-rich domain at the C-terminus. Accordingly, we designed the PAM sequence for CRISPR/Cas9 gene editing in a similar region of the fifth exon (Figure 1A). The homozygous *T_2_ crispr/dep1* mutants reserved the whole G gamma subunit protein domain but lost the cys-rich domain. Then, the yield-related traits of the *crispr/dep1* plants were investigated. The height of the *crispr/dep1* plants was significantly less than that of WT, and no obvious differences in the panicle number per plant and the setting rate were observed between WT and the mutant. As expected, the grain number per panicle of *crispr/dep1* was significantly increased, whereas the 1,000-grain weight of *crispr/dep1* was significantly decreased compared to WT. 

The premature termination of *GS3* at the G gamma protein domain causes a long-grain phenotype [17]. To imitate the elite allele of *GS3*, the sgRNA sequence was designed before the G gamma protein domain, to eliminate the entire G gamma protein domain, and to create the *crispr/gs3* mutants. The homozygous T_2_ *crispr/gs3* mutants exhibited an obvious increase in grain length, which significantly increased the 1,000-grain weight, whereas the grain number per panicle of the mutant was significantly decreased compared to that of WT. There were no significant differences in panicle number and setting rate between *crispr/gs3* and WT. Therefore, the *crispr/dep1* mutant showed improved grain yield due to the increased grain number per panicle, whereas the *crispr/gs3* mutant exhibited comparable yield performance to WT due to the decreased grain number per panicle, although the grain size was significantly increased. 

### 2.2. The Grain Changes of DEP1 and GS3 Mutants

**Figure 2:**
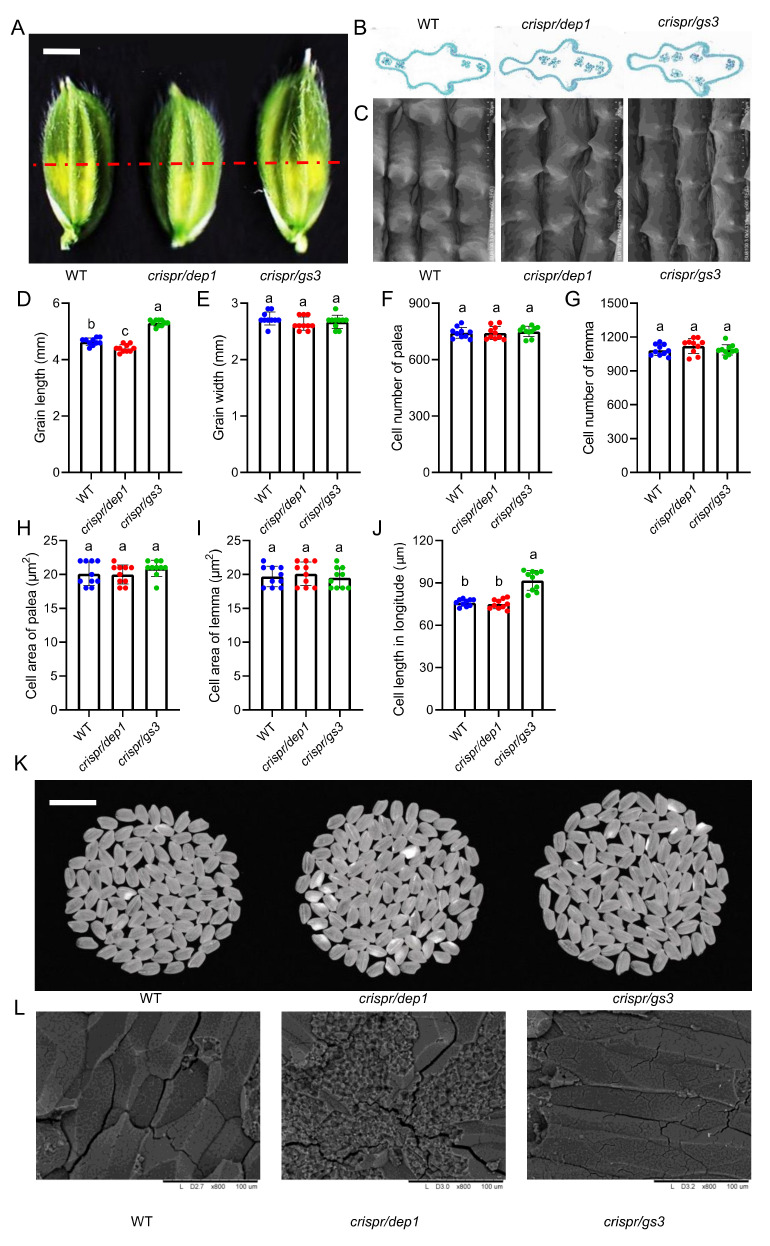
Histological analysis of spikelet hulls. (**A**) Young spikelet hulls of the *japonica* variety Sasanishiki (WT) and the *crispr/dep1* and *crispr/gs3* mutants. The red line indicates the position of the cross-section. Scale bar = 3 mm. (**B**) The cross-section of the spikelet hulls. Scale bar = 200 μm. (**C**) Scanning electron microscope analysis of the outer surfaces of the glumes. Scale bars = 100 μm. (**D**–**J**) The grain length (**D**), grain width (**E**), palea cell number (**F**), lemma cell number (**G**), palea cell area (**H**), lemma cell area (**I**), and cell length in longitude (**J**) of WT, *crispr/dep1*, and *crispr/gs3.* The data are the mean ± s.d. (n = 10 plants), and different letters indicate significant differences at the 5% level. The red, blue and green dots indicate the individual values of WT, *crispr/dep1*, and *crispr/gs3*, respectively. (**K**) The head rice of WT, *crispr/dep1*, and *crispr/gs3.* Scale bars = 1 cm. (**L**) Scanning electron microscope images of the transverse section of WT, *crispr/dep1*, and *crispr/gs3* starch granule. Scale bars = 100 μm.

**Figure 3:**
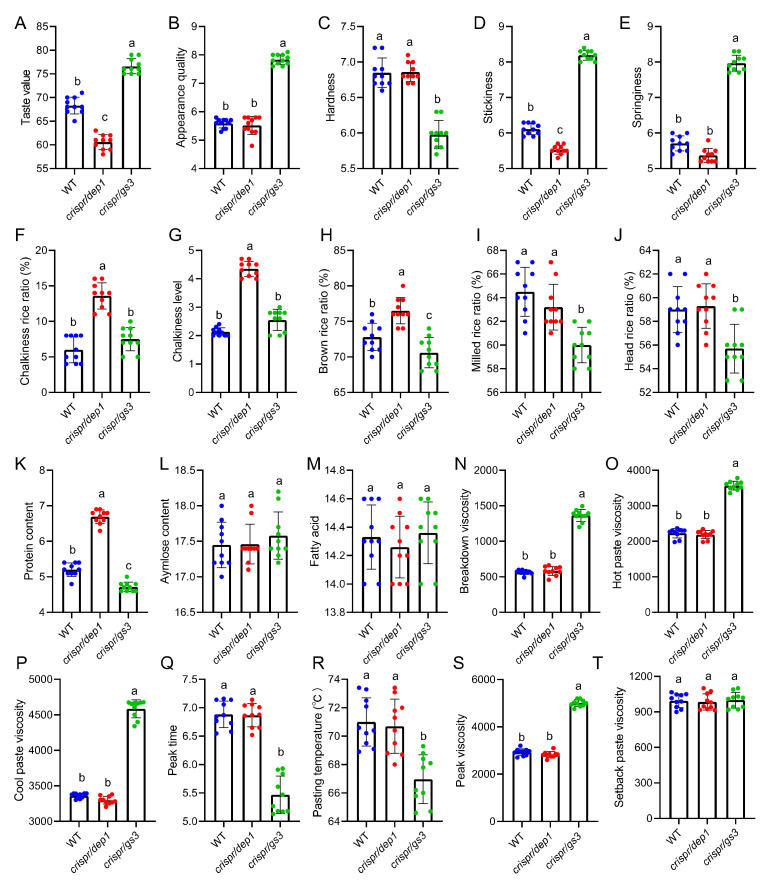
The quality traits of the *japonica* variety Sasanishiki (WT) and the gene-edited mutants. (**A–T**) The taste value, appearance quality, hardness, stickiness, springiness, chalkiness rice ratio (%), chalkiness level, brown rice ratio (%), milled rice ratio (%), head rice ratio (%), protein content, amylose content, fatty acid, breakdown viscosity, hot paste viscosity, cold paste viscosity, peak time, pasting temperature, peak viscosity, and setback paste viscosity. The data are the mean ± s.d. (n = 10 plants), and different letters indicate significant differences at the 5% level. The red, blue and green dots indicate the individual values of WT, *crispr/dep1*, and *crispr/gs3*, respectively.

The spikelet hull just before fertilization was much longer in *crispr/gs3* than in WT and *crispr/dep1*, and *crispr/gs3* had similar grain width to WT and *crispr/dep1* (Figure 2A,D,E). An investigation of a cross-section of the spikelet hulls showed that there were no obvious differences in spikelet perimeter, cell number, or cell area of both the palea and lemma between WT and the mutants (Figure 2B,F–I). In addition, an obvious difference in longitudinal cell density on the outer surface of the glume was observed between WT and the mutants (Figure 2C,J). Thus, these findings suggest that the long grain length in the spikelet hull was the result of an increase in longitudinal cell density on the outer surface of the glume. The milled rice from *crispr/gs3* was longer than that from WT and *crispr/dep1*. More importantly, the *cripsr/gs3* mutants could eliminate the negative effect of the transparency of the milled rice from *crispr/dep1*. Some milled rice from *crispr/dep1* showed an obvious white core or belly area in the endosperm, unlike that from WT and *crispr/gs3*, which showed no or very little chalkiness (Figure 2 and Figure 3). The scanning electron microscope images showed that the endosperm of WT and *crispr/gs3* was comprised of more tightly packed and sharp-edged polygonal starch granules (Figure 2L). Accordingly, *crispr/gs3* was assumed to confer better quality compared to that of WT and *crispr/dep1*.

### 2.3. The Quality Traits of WT and the DEP1 and GS3 Mutants

As expected, obvious differences in yield-related traits were observed between WT and the mutants. A comprehensive investigation of the quality traits of the *DEP1* and *GS3* mutants was subsequently conducted. A total of 20 quality traits including taste value, texture properties, milling quality, appearance quality, nutritional quality, and RVA profiles were investigated (Figure 3). The *crispr/gs3* mutant showed a strong advantage in taste value and appearance quality compared to WT and the *crispr/dep1* mutant (Figure 3A,B). Cooked rice texture properties such as hardness, stickiness, and springiness are appealing to consumers and directly reflect eating and cooking quality. The hardness of *crispr/gs3* was significantly decreased, whereas the stickiness and springiness were significantly increased compared to that of WT and *crispr/dep1*. There were no obvious differences in chalkiness-related traits between WT and *cirspr/gs3*, but the *crispr/dep1* mutant exhibited a dramatic increase in terms of both the chalkiness rice ratio and its chalkiness level. The milling quality of *crispr/gs3* was impaired due to elongated grain length compared to that of WT and *crispr/dep1*. There were no significant differences in amylose content or fatty acids among WT and the two mutants. The protein content of *crispr/dep1* was significantly increased, whereas the protein content of *crispr/gs3* was obviously decreased compared to that of WT. The RVA profiles revealed that the breakdown viscosity, hot paste viscosity, cool paste viscosity, peak viscosity, and setback paste viscosity of *crispr/gs3* were significantly increased, whereas the peak time and pasting temperature of *crispr/gs3* were significantly decreased compared to WT and *crispr/dep1*. There were no obvious differences in setback paste viscosity among WT and the two mutants. Overall, the *crispr/gs3* gene-edited plants exhibited an advantage in almost all quality traits, except for a slight decline in milling quality, whereas the *crispr/dep1* mutant exhibited comparable quality traits to WT despite the lower taste value and chalkiness related traits. 

### 2.4. Molecular Design Breeding of a “Super Rice” Variety

**Figure 4:**
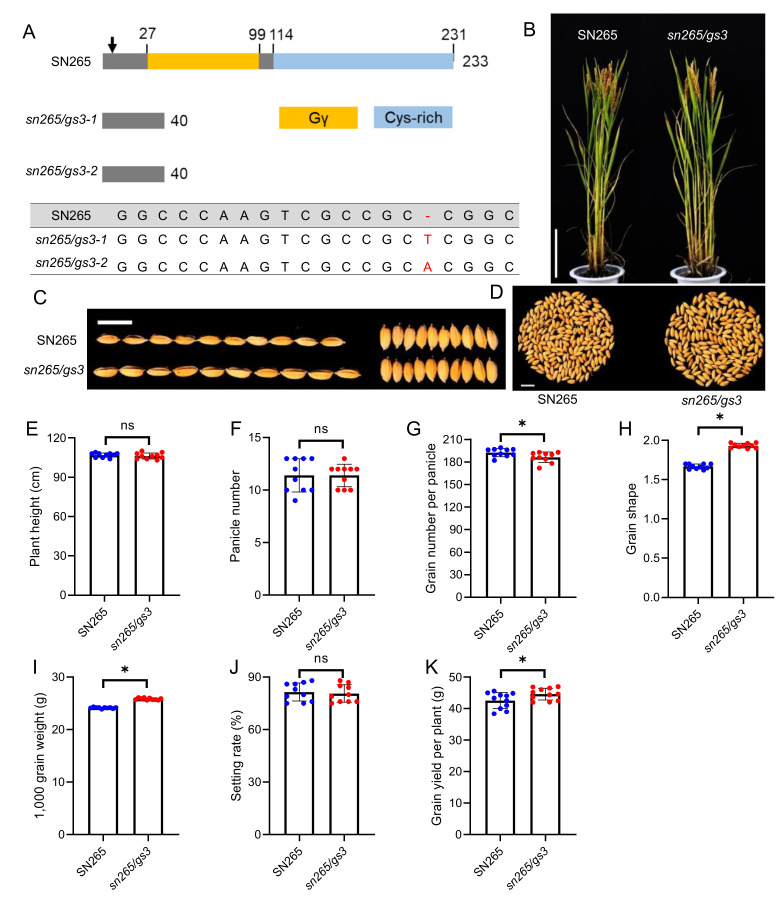
The yield-related traits of “super rice” variety SN265 and *gs3* gene-edited mutants. (**A**) Schematic diagram of the genomic region and functional domain of SN265 and the CRISPR gene-edited mutants. The sequence alignment of the sgRNA target region shows altered bases in different lines. The arrow indicates the position of the sgRNA (**B**) The plant architecture of SN265 and the *gs3* gene-edited mutants. Bar = 20 cm. (**C**) The grain size of SN265 and the *gs3* gene-edited mutants. Bar = 1 cm. (**D**) The grain number per panicle of SN265 and the gs3 gene edited mutants. Bar = 1 cm. (**E–K**) The plant height, panicle number, grain number per panicle, grain shape, 1000—grain weight, setting rate, and grain yield per plant of SN265 and the *gs3* gene-edited mutants. The data are the mean ± s.d. (n = 10 plants), and * indicates significant differences at the 5% level. The red, blue and green dots indicate the individual values of WT, *crispr/dep1*, and *crispr/gs3*, respectively.

Since the 1980s, several high-yielding *japonica* rice strains, whose architecture is characterized by dense and erect panicles, have been released as commercial varieties. In China, examples of this *japonica* ideotype, such as SN265, have dominated the *japonica* rice acreage. SN265 harbors a truncated allele of *dep1* and has high-yield potential in addition to preferable integrated traits, such as lodging resistance and a well-developed vascular system. However, the short and round grains caused by the truncated *dep1* allele and the mediocre eating and cooking quality have limited further commercial promotion. To enhance the quality traits of SN265, we conducted a molecular design breeding strategy by gene editing at the *GS3* locus of SN265 (Figure 4). The homozygous T_2_ *sn265/gs3* mutants showed similar plant architecture to SN265, and there were no significant differences in plant height, panicle number, and setting rate between SN265 and *sn265/gs3*. As expected, the grain length of *sn265/gs3* was significantly increased, which enhanced the 1000-grain weight, and eventually increased the grain yield per plant, although the grain number per panicle was significantly decreased compared to that of SN265.

### 2.5. The Quality Traits of the Improved Variety

**Figure 5:**
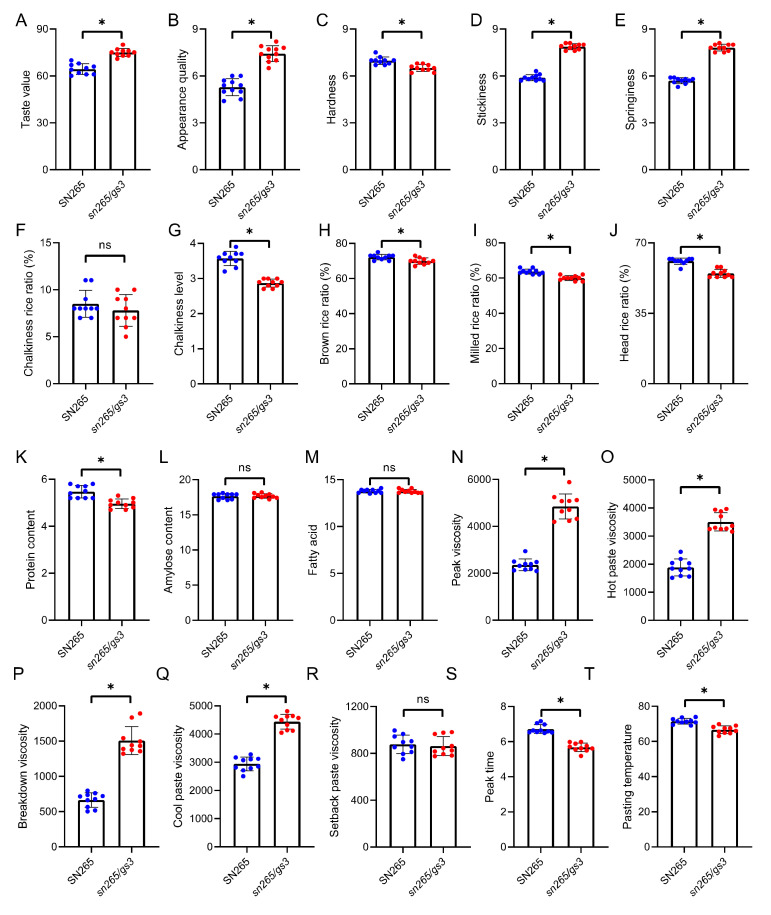
The quality traits of *japonica* variety SN265 (WT) and the *sn265/gs3* gene-edited mutant. (**A–T**) The taste value, appearance quality, hardness, stickiness, springiness, chalkiness rice ratio (%), chalkiness level, brown rice ratio (%), milled rice ratio (%), head rice ratio (%), protein content, amylose content, fatty acid, breakdown viscosity, hot paste viscosity, cold paste viscosity, peak time, pasting temperature, peak viscosity, and setback paste viscosity. The data are the mean ± s.d. (n = 10 plants), and * indicates significant differences at the 5% level. The blue and red dots indicate the individual values of SN265 and *sn265/gs3*, respectively.

To confirm that the molecular design breeding strategy resulted in enhanced quality traits while maintaining the yield potential of SN265, 20 quality traits of SN265 and *sn265/gs3* were surveyed (Figure 5). As expected, the taste value and appearance quality of *sn265/gs3* were obviously enhanced compared to those of SN265. The hardness of *sn265/gs3* was significantly decreased, whereas the stickiness and springiness increased significantly compared to SN265. There were no obvious differences in the chalkiness rice ratio between SN265 and *sn265/gs3*, but the chalkiness level of *sn265/gs3* was improved compared to SN265. SN265 showed an advantage in milling quality compared with *sn265/gs3*. Both SN265 and *sn265/gs3* showed similar amylose content and fatty acid content, but *sn265/gs3* had a lower value of protein content than that of SN265. Significant differences in RVA profiles were observed between SN265 and *sn265/gs3*, with the exception of setback paste viscosity. Taken together, the gene-editing of *GS3* could dramatically enhance the quality traits of SN265. 

## 3. Discussion

Recent molecular research has demonstrated that gamma subunits are considered important components of heterotrimeric G proteins that regulate multiple crucial growth and development processes [6]. *DEP1* and *GS3* represent type C G protein gamma subunits that are widespread throughout seed plants but do not exist in animals [6,11]. This type of C G protein subunit has a modular arrangement with a conventional plant-specific G gamma subunit protein domain at its N-terminus, followed by a cys-rich domain at the C-terminus. Numerous studies have revealed that the length variation in the cys-rich domain in *DEP1* and *GS3* contributes to the diversity in grain number and grain size [17,18,19]. Although the functions of *DEP1* and *GS3* in the regulation of yield potential have been extensively studied, the functions involved in grain quality remain unknown. Quality improvement is the ultimate goal of rice breeding. In this study, we found that the truncated *dep1* allele enhanced the yield potential by increasing the grain number per panicle, with a negative impact on taste value, protein content, and chalkiness-related traits. In contrast, the knockout *gs3* mutant showed a dramatic improvement in taste value, appearance quality, texture properties, and RVA profiles without a yield penalty. These results reveal an opportunity to simultaneously improve the yield and quality of rice through manipulating the two type C G protein gamma subunits. Our subsequent molecular design breeding of super rice variety SN265 confirmed that the combination of the elite alleles could enhance both yield and quality.

Heterotrimeric G proteins consist of G alpha, G beta, and G gamma units. The rice genome encodes one G alpha protein, one G beta protein, and five G gamma proteins. By combining different G protein variants constructed by CRISPR/Cas9 (ko), gene over-expression (OE), and RNA-interference (Ri), grain length can be increased by up to 19% or decreased by up to 35%, which has produced a 28% increase to a 40% decrease in grain weight in previous research [20]. The cross between *GS3Ri* plants and *dep1OE* plants showed that the *GS3Ri*/*dep1OE* plants exhibited reduced grain length, similar to *dep1OE* transgenic plants. The cross between *GS3^ko^* and *DEP1^ko^* demonstrated that the *GS3^ko^* mutant exhibited increased grain size, the *DEP1^ko^* mutant had reduced grain length, and the grain length of the *GS3^ko^DEP1^ko^* double mutant was intermediate between those of the *GS3^ko^* and *DEP1^ko^* single mutants [20]. The results of the present study confirmed that the *gs3* mutant exhibited increased grain length, and the *gs3dep1* double mutant showed intermediate grain length compared to that of the single mutants. However, in the present study, the *dep1* mutant showed a similar grain length to WT, in contrast with the findings of a previous study where *DEP1^ko^* exhibited reduced grain length [19]. The difference in phenotype might be due to the different positions of the sgRNA in CRISPR/Cas9 gene editing. The present study generated a truncated *dep1* allele, which is similar to natural variations, such as SN265 [11], and Sun et al. designed the sgRNA at the first exon of *DEP1*, which eliminated both the G gamma subunit domain and the cys-rich domain. The latter mutant may have a more severe impact on grain length. Taken together, not only can *DEP1* and *GS3* be used to predictably design grain size, but they can also be used to enhance taste value, appearance quality, texture properties, and RVA profiles. This paper presents a strategy for the simultaneous improvement of grain yield and quality through the manipulation of two type C G protein gamma subunits in rice. Thus, the combination of other G protein subunits besides *DEP1* and *GS3* might provide more preferable germplasms with high grain yield and quality.

## 4. Materials and Methods

### 4.1. Plant Materials

In this study, Sasanishiki (WT) and homozygous T_2_ mutants of *DEP1* and *GS3* were employed. Shenyang Agricultural University’s Rice Research Institute (N41°, E123°) was used to conduct field tests. The seeds were planted on 21 May 2021 after being sowed on 15 April. Each line was planted in three rows, with 10 plants per row and a 30 cm × 13.3 cm plant spacing. Fertilizers were applied at a rate of 150 kg N per hectare, 150 kg P per hectare, and 150 kg K per hectare as a basal dressing. As a top application, 75 kg N per acre was applied 7 days after transplanting. After 45 days of heading for each line, the paddies were harvested.

### 4.2. Vector Construction and Plant Transformation

The CRISPR/Cas9 gene-editing vector construction was carried out as previously described [21]. The 23-bp targeting sequences (including PAM) inside the target genes were selected, and a BLAST search was performed against the Nipponbare genome to ensure targeting specificity [22]. The rice transformation was carried out following the procedure described in an earlier report [23]. Genomic DNA was collected from these transformants after rice transformation. Primer pairs around the intended target site were used in the PCR amplification. The PCR products (300–500 bp) were sequenced using the degenerate sequence decoding technique [24].

### 4.3. Microscopy Observations 

Fresh young spikelet hulls were fixed, dehydrated, and embedded in Paraplast Plus (P3683-1KG, Sigma-Aldrich, St. Louis, Missouri, USA), after which they were sliced into 10-m-thick slices. Light microscopy (CX43, Olympus, Tokyo, Japan) was used to examine cross-sections, and ImageJ and Adobe Photoshop CS2 software were used to count and measure the number of cells in the outer parenchyma cell layer of hulls. The exterior surfaces of the spikelet glumes were studied using a scanning electron microscope (S-4800, Hitachi, Tokyo, Japan). Natural cross-sections of mature milled rice were examined using a scanning electron microscope (SEM TM1000, Hitachi, Tokyo, Japan) to look for starch granules.

### 4.4. Quality Traits Measurement 

The plants were harvested from the middle rows for the investigation of yield and quality traits. After harvest, mature rice grains were milled, air dried and stored at room temperature for three months. Then the brown rice ratio, milled rice ratio, and head rice ratio were calculated. The brown rice ratio was evaluated after the grains were dehulled using a Rubber Roll Sheller (THU testing hunsker, Satake, Hiroshima, Japan). The brown rice was then milled using rice-polishing equipment (TM05 test mill, Satake, Hiroshima, Japan). The amylose content and protein content were surveyed according to The National Standard of the People’s Republic of China (GB/T17891–1999). The rice protein compositions were extracted and measured using the methods described by Tan et al. (1999) [25]. The pasting properties of the rice flour were investigated using a Rapid Visco Analyser (Tech Master, Newport Scientific, Warriewood, Australia), and the data were analyzed according to the experimental procedure described by Zhang et al. (2013) [26].

## Data Availability

The study did not report any data.

## References

[1] Muthayya S., Sugimoto J.D., Montgomery S., Maberly G.F. (2014). An overview of global rice production, supply, trade, and consumption. Ann. N. Y. Acad. Sci..

[2] Fei C., Xu Q., Xu Z., Chen W. (2020). Effect of Rice Breeding Process on Improvement of Yield and Quality in China. Rice Sci..

[3] Urano D., Miura K., Wu Q., Iwasaki Y., Jackson D., Jones A.M. (2016). Plant Morphology of Heterotrimeric G protein Mutants. Plant Cell Physiol..

[4] Temple B.R., Jones A.M. (2007). The plant heterotrimeric G-protein complex. Annu. Rev. Plant Biol..

[5] Jones A.M., Assmann S.M. (2004). Plants: The latest model system for G-protein research. EMBO Rep..

[6] Xu Q., Zhao M., Wu K., Fu X., Liu Q. (2016). Emerging Insights into Heterotrimeric G Protein Signaling in Plants. J. Genet. Genom..

[7] Ferrero-Serrano A., Assmann S.M. (2016). The alpha-subunit of the rice heterotrimeric G protein, RGA1, regulates drought tolerance during the vegetative phase in the dwarf rice mutant d1. J. Exp. Bot..

[8] Jangam A.P., Pathak R.R., Raghuram N. (2016). Microarray Analysis of Rice d1 (RGA1) Mutant Reveals the Potential Role of G-Protein Alpha Subunit in Regulating Multiple Abiotic Stresses Such as Drought, Salinity, Heat, and Cold. Front. Plant Sci..

[9] Utsunomiya Y., Samejima C., Takayanagi Y., Izawa Y., Yoshida T., Sawada Y., Fujisawa Y., Kato H., Iwasaki Y. (2011). Suppression of the rice heterotrimeric G protein beta-subunit gene, RGB1, causes dwarfism and browning of internodes and lamina joint regions. Plant J..

[10] Miao J., Yang Z., Zhang D., Wang Y., Xu M., Zhou L., Wang J., Wu S., Yao Y., Du X. (2019). Mutation of RGG2, which encodes a type B heterotrimeric G protein gamma subunit, increases grain size and yield production in rice. Plant Biotechnol. J..

[11] Trusov Y., Chakravorty D., Botella J.R. (2012). Diversity of heterotrimeric G-protein γ subunits in plants. BMC Res. Notes.

[12] Huang X., Qian Q., Liu Z., Sun H., He S., Luo D., Xia G., Chu C., Li J., Fu X. (2009). Natural variation at the DEP1 locus enhances grain yield in rice. Nat. Genet..

[13] Xu H., Zhao M., Zhang Q., Xu Z., Xu Q. (2016). The DENSE AND ERECT PANICLE 1 (DEP1) gene offering the potential in the breeding of high-yielding rice. Breed. Sci..

[14] Botella J.R. (2012). Can heterotrimeric G proteins help to feed the world?. Trends Plant Sci..

[15] Zhao M., Sun J., Xiao Z., Cheng F., Xu H., Tang L., Chen W., Xu Z., Xu Q. (2016). Variations in DENSE AND ERECT PANICLE 1 (DEP1) contribute to the diversity of the panicle trait in high-yielding japonica rice varieties in northern China. Breed. Sci..

[16] Fan C., Xing Y., Mao H., Lu T., Han B., Xu C., Li X., Zhang Q. (2006). GS3, a major QTL for grain length and weight and minor QTL for grain width and thickness in rice, encodes a putative transmembrane protein. Theor. Appl. Genet..

[17] Mao H., Sun S., Yao J., Wang C., Yu S., Xu C., Li X., Zhang Q. (2010). Linking differential domain functions of the GS3 protein to natural variation of grain size in rice. Proc. Natl. Acad. Sci. USA.

[18] Li X., Tao Q., Miao J., Yang Z., Gu M., Liang G., Zhou Y. (2019). Evaluation of differential qPE9-1/DEP1 protein domains in rice grain length and weight variation. Rice.

[19] Li M., Li X., Zhou Z., Wu P., Fang M., Pan X., Lin Q., Luo W., Wu G., Li H. (2016). Reassessment of the Four Yield-related Genes Gn1a, DEP1, GS3, and IPA1 in Rice Using a CRISPR/Cas9 System. Front. Plant Sci..

[20] Sun S., Wang L., Mao H., Shao L., Li X., Xiao J., Ouyang Y., Zhang Q. (2018). A G-protein pathway determines grain size in rice. Nat. Commun..

[21] Li W., Zhu Z., Chern M., Yin J., Yang C., Ran L., Cheng M., He M., Wang K., Wang J. (2017). A Natural Allele of a Transcription Factor in Rice Confers Broad-Spectrum Blast Resistance. Cell.

[22] Hsu P.D., Scott D.A., Weinstein J.A., Ran F.A., Konermann S., Agarwala V., Li Y., Fine E.J., Wu X., Shalem O. (2013). DNA targeting specificity of RNA-guided Cas9 nucleases. Nat. Biotechnol..

[23] Nishimura A., Aichi I., Matsuoka M. (2006). A protocol for Agrobacterium-mediated transformation in rice. Nat. Protoc..

[24] Ma X., Zhang Q., Zhu Q., Liu W., Chen Y., Qiu R., Wang B., Yang Z., Li H., Lin Y. (2015). A robust CRISPR/Cas9 system for convenient, high-efficiency multiplex genome editing in monocot and dicot plants. Mol. Plant.

[25] Tan Y.F., Li J.X., Yu S.B., Xing Y.Z., Xu C.G., Zhang Q. (1999). The three important traits for cooking and eating quality of rice grains are controlled by a single locus in an elite rice hybrid, Shanyou 63. Theor. Appl. Genet..

[26] Zhang C.-Q., HU B., Zhu K.-Z., Zhang H., Leng Y.-L., Tang S.-Z., Gu M.-H., Liu Q.-Q. (2013). QTL Mapping for Rice RVA Properties Using High-Throughput Re-sequenced Chromosome Segment Substitution Lines. Rice Sci..

